# Prognostic value of programmed cell death ligand 1 (PD-L1) for hepatocellular carcinoma: a meta-analysis

**DOI:** 10.1042/BSR20200459

**Published:** 2020-04-17

**Authors:** Xiao-Song Li, Jun-Wei Li, Hui Li, Tao Jiang

**Affiliations:** 1 Department of Hepatobiliary-Pancreatic Surgery, China-Japan Union Hospital of Jilin University, Changchun 130000, Jilin, China; 2 Department of Neurosurgery, China-Japan Union Hospital of Jilin University, Changchun, China

**Keywords:** clinicopathology, HCC, meta-analysis, PD-L1, prognosis, survival

## Abstract

The prognostic role of programmed death ligand-1 (PD-L1) expression in hepatocellular carcinoma (HCC) has been widely studied but the results are controversial. In this comprehensive meta-analysis, we elucidated the clinical value of PD-L1 in HCC. Relevant studies were systematically searched in the Cochrane Library, EMBASE, and PubMed until June 27, 2019. Eligible studies were validated for the prognostic effect of PD-L1 on the overall survival (OS), disease-free survival (DFS), and relapse-free survival (RFS) in HCC using a hazard ratio (HR) and its 95% confidence interval (95% CI). Twenty-three studies with 3529 patients were involved in this meta-analysis. The pooled results revealed that high membrane-bound PD-L1 (mPD-L1) expression was associated with poor OS (HR: 1.42; 95% CI: 1.12–1.80; *P* = 0.004) and had no significant correlation with RFS (HR: 1.14; 95% CI: 0.85–1.54; *P* = 0.39), and DFS (HR: 1.36; 95% CI: 0.81–2.28; *P* = 0.25). The results also indicated that high soluble PD-L1 (sPD-L1) levels were associated with worse OS (HR: 2.93; 95% CI: 2.20–3.91; *P* < 0.00001). In addition, high mPD-L1 expression was associated with high alpha-fetoprotein levels (AFP; OR = 1.46; 95% CI: 1.16–1.84; *P* = 0.001), hepatitis (OR = 0.72; 95% CI: 0.54–0.98; *P* = 0.03), poor tumor differentiation (OR = 0.68; 95% CI: 0.55–0.84; *P* = 0.03), and tumor-infiltrating lymphocytes (OR = 3.39; 95% CI: 1.06–10.91; *P* = 0.04). The mPD-L1 expression had no significant correlation with age, number of tumors, gender, tumor size, liver cirrhosis, vascular invasion, tumor encapsulation, or TNM stage. The study revealed that high mPD-L1 expression in the tumor tissue and high sPD-L1 levels were associated with shorter OS in HCC. Moreover, overexpression of mPD-L1 was significantly associated with poor tumor differentiation, hepatitis, AFP elevation, and tumor-infiltrating lymphocytes.

## Introduction

Hepatocellular carcinoma (HCC) is one of the most common cancers worldwide and ranks as the second most prevalent cause of cancer-related mortality [1]. About one-third of the patients with an early stage of HCC receive curative treatment such as surgical resection, liver transplantation, and ablation, while the remaining patients with advanced HCC receive palliative treatment. Viral infection is one of the main causes of liver cirrhosis and HCC [1]. After a chronic viral infection, persistent innate and adaptive immune responses lead to immune tolerance and tumor formation. The programmed cell death-1 (PD-1)/programmed cell death ligand 1 (PD-L1) pathway plays a critical role in the development of chronic infection, in escaping from tumor immune response, and tumor microenvironment formation [2]. PD-1 is a member of the CD28 family and has two ligands PD-1 (PD-L1) and PD-L2 (also known as B7-H1 and B7-DC), which have the characteristics of costimulatory molecules [3]. In cell-mediated immunity, T-cell receptors are activated to recognize the tumor antigen expressed by tumor cell MHC molecules, and perforin and granzyme are released to attack tumor cells. At the same time, activated T cells release IFN-γ and other cytokines. Under the action of these cytokines, cancer cells up-regulate the expression of PD-L1, making it combine with PD-1 on T cells, weakening the attack of T cells. Tumor cells use a variety of immunosuppressive methods to resist tumor immunity, one of them is the PD-1/PD-L1 axis also known as the ‘immune checkpoint’ [4]. After PD-L1 combines with PD-1, it activates the PD-1/PD-L1 pathway that reduces the anti-tumor activity of T cells and induces T-cell apoptosis. This could be one of many mechanisms responsible for immune tolerance. PD-1 / PD-L1 pathway molecules include membrane-bound PD-1 / PD-L1 (mPD-1/ mPD-L1) and soluble PD-1 / PD-L1 (sPD-1 / sPD-L1). All these molecules play an important immunosuppressive role in tumor T-cell immune response [5–7]. However, their specific roles in tumor microenvironment are different. The role of sPD-L1 in tumor immunity needs further evaluation. It has been found that sPD-L1 could have other sources besides hydrolysis of mPD-L1 [8]. Recently, many studies have found that PD-L1 is related to tumor immunity and overexpression in various solid tumors such as colorectal cancer [9,10], pancreatic cancer [11], lung cancer [12], and melanoma [13]. Similarly, a number of studies have been carried on to examine the association between PD-L1 expression and prognosis of HCC; however, the results have been conflicting [14–17]. In addition, the authors of previous meta-analyses simply taken mPD-L1 and sPD-L1 as one indicator and not studied them separately. The present study aimed to pool the data of various studies to determine the association between PD-L1 expression (sPD-L1 and mPD-L1 separately) and the clinicopathological features and prognosis of HCC.

## Materials and methods

### Search strategy

We searched the Cochrane Library, EMBASE, and PubMed from its inception until June 27, 2019, using the keywords (‘hepatocellular carcinoma’ OR ‘hepatocarcinoma’ OR ‘hepatomas’ OR ‘liver carcinoma’ OR ‘liver cell carcinoma’ OR ‘HCC’ OR ‘liver cancer’) AND (‘programmed cell death-ligand 1’ OR ‘PD-L1’ OR ‘B7-H1’).

### Selection criteria

The studies satisfying the following criteria were included: (1) all patients were pathologically confirmed to have HCC; (2) tests showed expression of PD-L1 in tumor tissue soluble PD-L1 levels; (3) enough data were available to analyze the relationship between PD-L1 expression, and clinicopathological characteristics and prognosis; and (4) the manuscript should be originally published in English. The exclusion criteria are as follows: (1) case report, review, meta-analysis, and ongoing research; (2) animal experiments on HCC and research involving other tumors; (3) evidence insufficient to show the relationship between PD-L1 expression and survival prognosis; and (4) the samples tested are non-tumor tissues.

### Data extraction and quality assessment

Two authors (X.S.L. and J.W.L.) independently extracted the first author’s name, publication year, country, clinicopathological characteristics, PD-L1 (test specimen, test method, cut-off value, number of cases with positive expression of PD-L1), follow-up time, OS, DFS, and RFS (HR and 95% CI). According to the Newcastle−Ottawa quality assessment (NOS), two authors independently evaluated the quality of the eligible articles. NOS includes the following three quality parameters: selection, comparability, and the exposure. Any differences between the researchers were resolved through discussion and analysis.

### Statistical methods

All data in this meta-analysis were analyzed when using Revman 5.3 software (Revman, Cochrane Collaboration) and Stata 13.0 software (station, College Station), and *P* value < 0.05 was considered to be statistically significant. Heterogeneity between studies was evaluated by using the Cochrane *Q*-test and *P*-value. The data were said to be heterogenous if the chi-square *P*-value was less than 0.1 or *I*^2^ statistics was greater than 50%. If the heterogeneity was significant, random-effect model was adopted; or else, used the fixed-effect model. The potential publication bias was evaluated by a Begg’s funnel chart (*P* < 0.05). A sensitivity analysis was used to assess the source of heterogeneity in the pooled analysis by omitting one study at a time.

## Results

### Study characteristics

**Figure 1:**
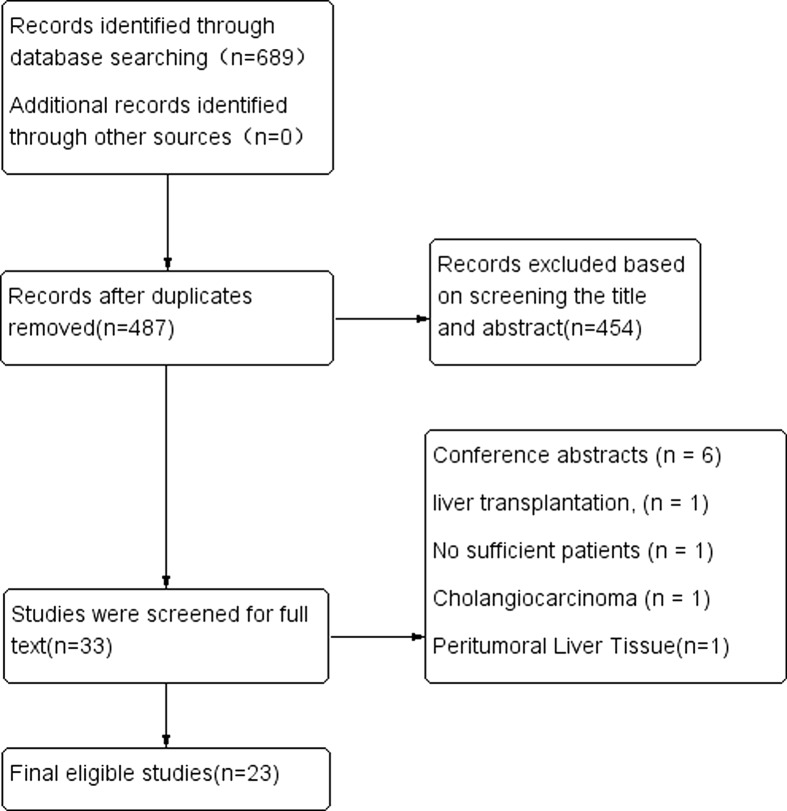
Flow diagram showing the study selection process followed in this meta-analysis

On initial screening, 689 studies were identified from three databases. After excluding 202 duplicate records, 487 studies were screened for titles and abstracts, and 33 relevant articles were screened for full texts. After a detailed study, 10 studies were excluded due to the following reasons: conference abstracts (*n* = 6); liver transplantation (*n* = 1); insufficient patients (*n* = 1); cholangiocarcinoma (*n* = 1); and the peritumoral liver tissue was tested (*n* = 1). Finally, 23 articles were included with a NOS score greater than 6 (Figure 1). Eighteen studies determined the mPD-L1 expression in tumor tissues. Among them, 16 studies analyzed the relationship between mPD-L1 expression and OS [14–29], seven studies analyzed the relationship between mPD-L1 expression and DFS [14,21,23,25,26,30,31], and six studies analyzed the relationship between mPD-L1 expression and RFS [15,19,20,22,27,28]. Besides, only three studies were conducted in Western countries [20,24,31]. The remaining 16 studies were conducted in Asia, of which 12 studies were from China [14,16–19,21,22,25–29]. In particular, Dai et al. [26] analyzed data from two independent groups, and both of them were included in this meta-analysis. There were five studies analyzing the relationship between the soluble PD-L1 levels and OS [32–36]. Studies didn’t report HR and 95% CIs directly. We used Kaplan–Meier curve to calculate them. Detailed clinicopathological data are shown in Tables 1 and 2.

**Table 1:** Characteristics of eligible studies involving the mPD-L1 expression in tumor tissue

Study	Country	Sample size	Stage	Method	Case number (High/Low)	Cut-off	Outcome	Follow-up time
Gao et al. [14]	China	240	I–III	IHC, IPP software	60/180	>75%	OS; DFS	Median time 60.8 month
Wu et al. [18]	China	71	I–IV	IHC, IPP software	35/36	Median	OS	80 months
Umemoto et al. [15]	Japan	80	I–IV	IHC, intensity of the staining scoring	37/43	Score 3	RFS; OS	Median time 2427 days
Kan et al. [16]	China	128	I–IV	IHC, positive cell rate	105/23	>25%	OS	Median time 10 months
Chen et al. [19]	China	217	I–III	IHC, positive cell rate	52/165	>5%	RFS, OS	>5 years
Gabrielson et al. [20]	America	58	I–IV	IHC, intensity and distribution	19/39	*N*	RFS, OS	84 months
Xie et al. [21]	China	90	I–III	IHC, positive cell rate	15/75	75%	OS; DFS	150 months
Chang, H. et al. [30]	Korea	146	T1–T4	IHC, slide scanner for positive cell rate	80/60	Median	DFS	153 months
Dai et al. [26]	China	90	N	IHC; Staining intensity	37/53	Score 2–3	OS; DFS	72 months
Dai et al.* [26]	China	90	N	IHC; Staining intensity	44/46	Score 2–3	OS; DFS	72 months
Jung et al. [23]	Korea	85	I–IV	IHC, staining percentage and intensity scoring	23/62	3–5	OS; DFS	125 months
Semaan et al. [24]	Germany	176	T1–T4	IHC, Tissue studio v.2.1 semiquantitative software	88/88	Median	OS	60 months
Sideras et al. [31]	Netherlands	146	N	IHC, staining intensity scoring	121/25	Cut-off with the low- est-2 log likelihood	DFS	>100 months
Chang, B. et al. [25]	China	145	N	IHC, positive cell rate	40/105	>5%	OS; DFS	120 months
Hu et al. [27]	China	136	I–III	IHC, positive cell rate of membranous staining	26/110	>1%	RFS, OS	60 months
Liu et al. [28]	China	453	I–IV	IHC, positive cell rate	87/366	≥5%	RFS, OS	132 months
Pei et al. [17]	China	143	N	IHC, positive cell rate	19/124	>1%	OS	87 months
Huang et al. [22]	China	411	I–III	IHC, positive cell rate	78/333	>5%	RFS, OS	1000 months
Zhang et al. [29]	China	46	I–IV	IHC, positive cell rate of membranous staining	21/25	2–3	OS	5 years

Dai * included data from Dai et al. (25) control group. Dai data from Dai et al. (25) experimental cohort.

**Table 2:** Characteristics of eligible studies involving the soluble PD-L1 levels

Study	Country	Sample size	Stage	Method	Case number (High/Low)	Cut-off	Outcome	Follow-up time
Zeng et al. [32]	China	109	I–III	Flow cytometric analysis	N	median	OS	36 months
Finkelmeier et al. [33]	Germany	215	I–IV	ELISA	63/152	0.8 ng/ml	OS	1464 days
Chang, B et al. [35]	China	120	I–II	Antibody array assay	35/85	11.2 µg/ml	OS;DFS	1600 days
Han et al. [36]	China	81	I–IV	ELISA	37/43	5.471 ng/ml	OS;DFS	120 mouths
Kim et al. [34]	Korea	53	I–IV	ELISA	43/10	1.315pg/ml	OS	72 months

### Survival analysis

**Figure 2:**
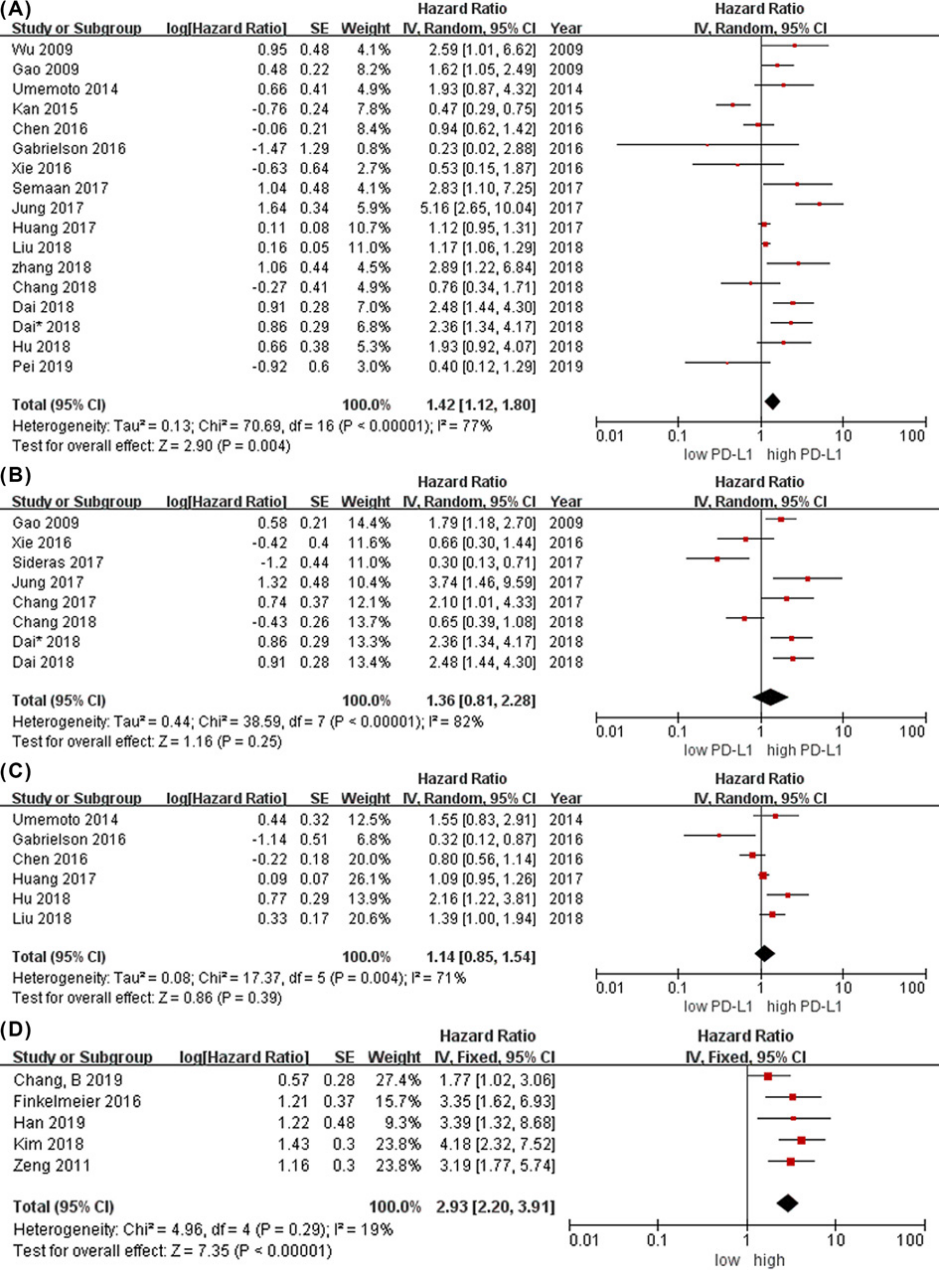
Meta-analysis of PD-L1 and prognosis in HCC (**A**) Forest plot between mPD-L1 and overall survival (OS). (**B**) Forest plot between mPD-L1 and disease free survival (DFS). (**C**) Forest plot between mPD-L1 and relapse free survival (RFS). (**D**) Forest plot between the soluble PD-L1 levels and overall survival (OS).

The pooled data of 17 studies indicated that overexpression of mPD-L1 was associated with shorter OS (HR: 1.42; 95% CI: 1.12–1.80; *P* = 0.004) with significant heterogeneity (*P* < 0.00001; *I*^2^ = 77%; Figure 2A). However, the pooled HR found no significant association between mPD-L1 expression and DFS (HR: 1.36; 95% CI: 0.81–2.28; *P* = 0.25) with heterogeneity (*P* < 0.00001; *I*^2^ = 82%; Figure 2B). Six studies with RFS data demonstrated no significant relationship between the expression of mPD-L1 and RFS (HR: 1.14; 95% CI: 0.85–1.54; *P* = 0.39) with significant heterogeneity (*P* = 0.004; *I*^2^ = 71%; Figure 2C). The soluble PD-L1 levels was associated with significantly shorter OS (HR: 2.93; 95% CI: 2.20–3.91; *P* < 0.00001) without significant heterogeneity (*P* = 0.29; *I*^2^ = 19%; Figure 2D).

### Association between mPD-L1 expression in the tumor tissue and clinicopathological features of HCC

**Figure 3:**
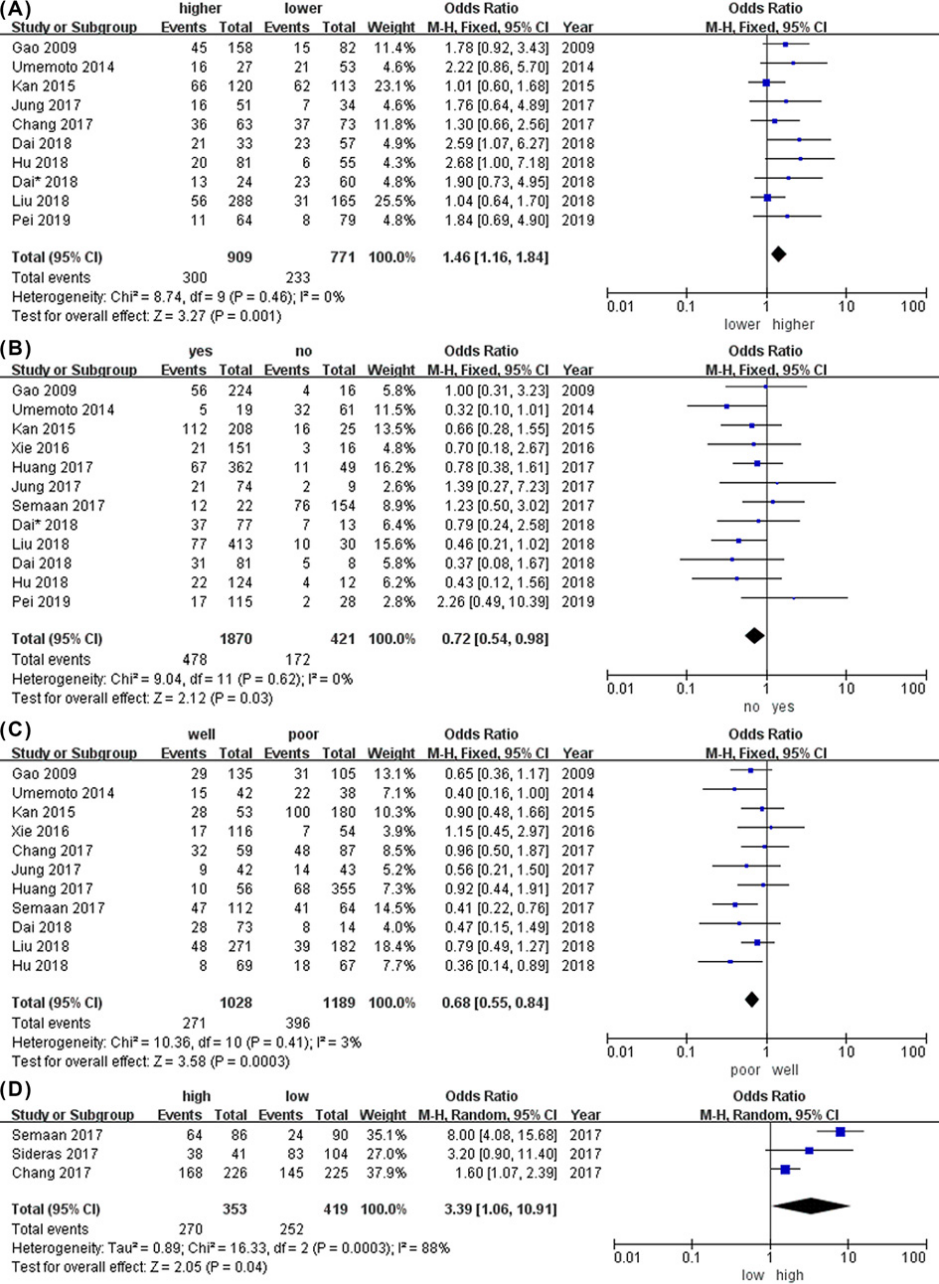
The association between mPD-L1 and clinicopathological features in HCC (**A**) Forest plot of HR for alpha-feto protein (AFP) levels. (**B**) Forest plot of HR for hepatitis history. (**C**) Forest plot of HR for tumor differentiation. (**D**) Forest plot of HR for CD8 + TILs.

Table 3 demonstrates the relationship between mPD-L1 expression and clinicopathological features. We found that high expression of mPD-L1 correlated with AFP (OR = 1.46; 95% CI: 1.16–1.84; *P* = 0.001), history of hepatitis (OR: 0.72; 95% CI: 0.54–0.98; *P* = 0.03), tumor differentiation (OR = 0.68; 95% CI: 0.55–0.84; *P* = 0.03), and tumor-infiltrating lymphocytes (OR: 3.39; 95% CI: 1.06–10.91; *P* = 0.04; Figure 3A–D). However, the high expression was demonstrated not significantly correlated with age, sex, tumor size, liver cirrhosis, vascular invasion, number of tumors, tumor encapsulation, or TNM stage.

**Table 3:** Association between high mPD-L1 and clinicopathological features

Characteristics	Studies	Cases	Odds ratio (95% CI)	*P*	Heterogeneity	
					*I*²	*P*
Age (>50 vs ≤50)	10	1734	1.17 [0.94, 1.45]	0.17	0%	0.54
Gender (male/female)	13	2345	1.22 [0.93, 1.62]	0.16	12%	0.32
AFP (high/low)	10	1680	1.46 [1.16, 1.84]	0.001	0%	0.46
Tumor size (<5 vs ≥ 5)	10	2015	0.82 [0.67, 1.02]	0.07	40%	0.09
Liver cirrhosis (yes/no)	8	1105	0.99 [0.58, 1.69]	0.96	61%	0.01
Hepatitis (yes/no)	12	2291	0.72 [0.54, 0.98]	0.03	0%	0.62
Tumor number (multiple/single)	8	1634	0.92 [0.69, 1.22]	0.54	40%	0.11
Vascular invasion (yes/no)	7	1558	0.99 [0.55, 1.78]	0.97	73%	0.001
TNM stage (advanced/early)	9	1860	1.23 [0.97, 1.56]	0.05	48%	0.09
Differentiation (well/poor)	11	2217	0.68 [0.55, 0.84]	0.0003	3%	0.41
CD8+ TILs (high/low)	3	772	3.39 [1.06, 10.91]	0.04	88%	0.0003
Tumor encapsulation (no/yes)	3	787	0.97 [0.66, 1.42]	0.86	79%	0.009

### Publication bias and sensitivity analyses

**Figure 4:**
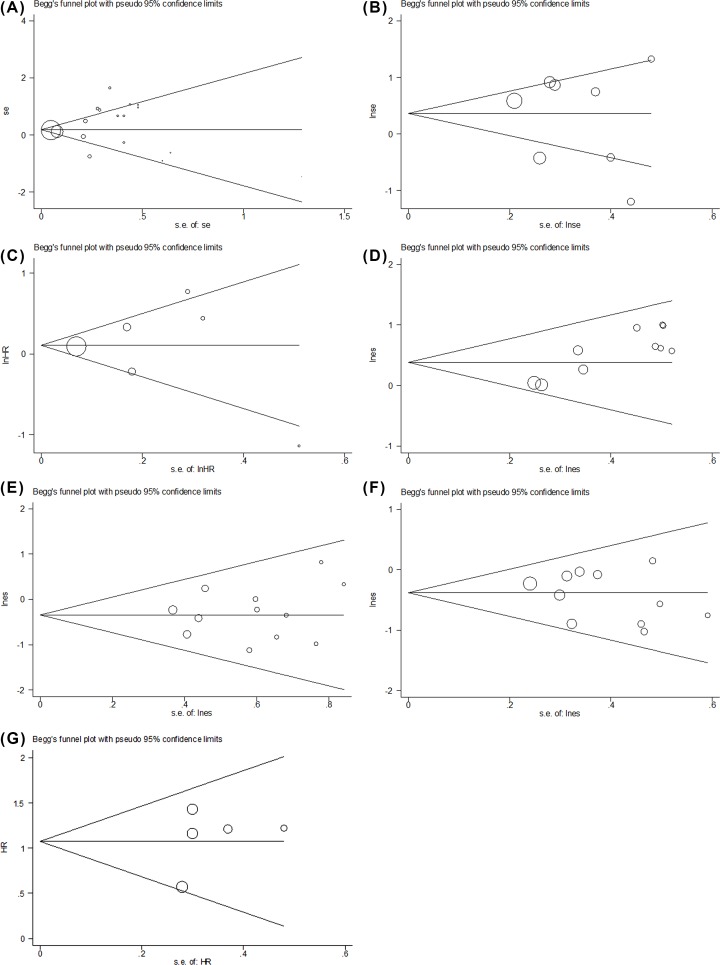
Begg’s funnel plot for publication bias test (**A**) Begg’s funnel plot for publication bias in studies reporting OS and mPD-L1; *P* = 0.536. (**B**) Begg’s funnel plot for publication bias in studies reporting DFS and mPD-L1; *P* = 0.711. (**C**) Begg’s funnel plot for publication bias in studies reporting RFS and mPD-L1; *P* = 0.707. (**D**) Begg’s funnel plot for publication bias in studies reporting AFP levels and mPD-L1; *P* = 0.283. (**E**) Begg’s funnel plot for publication bias in studies reporting hepatitis history and mPD-L1; *P* = 0.537. (**F**) Begg’s funnel plot for publication bias in studies reporting tumor differentiation and mPD-L1; *P* = 0.755. (**G**) Begg’s funnel plot for publication bias in studies reporting OS and sPD-L1; *P* = 0.564.

**Figure 5:**
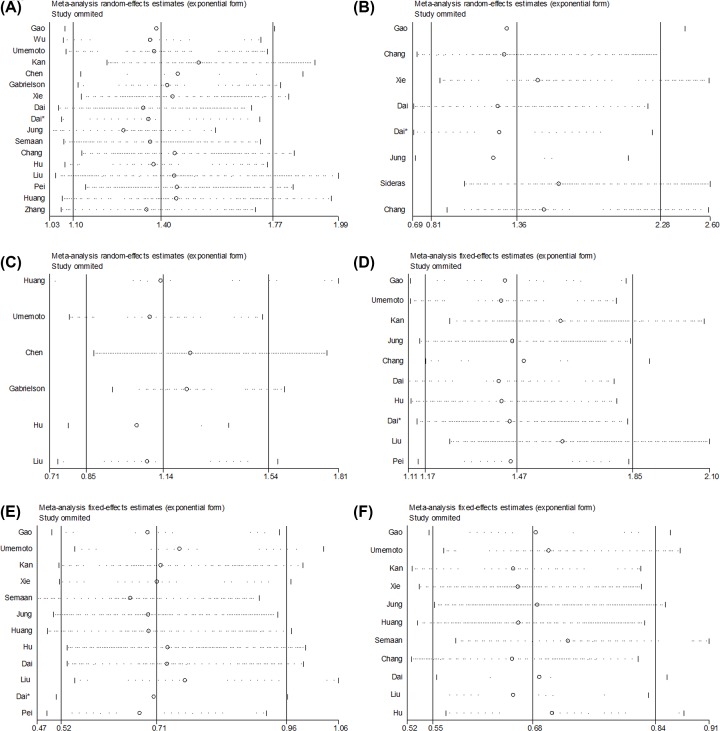
Sensitivity analysis of this meta-analysis (**A**) Sensitivity analysis for OS and mPD-L1. (**B**) Sensitivity analysis for DFS and mPD-L1. (**C**) Sensitivity analysis for RFS and mPD-L1. (**D**) Sensitivity analysis for AFP and mPD-L1. (**E**) Sensitivity analysis for hepatitis history and mPD-L1. (**F**) Sensitivity analysis for tumor differentiation and mPD-L1.

No evidence for apparent publication bias for the analysis between mPD-L1 and OS was found (Begg’s test: *P* = 0.536; Figure 4A). Likewise, no apparent publication bias was found for RFS and DFS analysis (Figure 4B,C). Meanwhile, for the analysis of clinicopathological features (Figure 4D–F), we found no obvious publication bias. Additionally, there was no apparent publication bias for analysis involving sPD-L1 (Figure 4G). Sensitivity analysis showed that none of the studies remarkably affected the combined HR for OS, DFS, RFS, AFP, tumor differentiation, and hepatitis (Figure 5A–F). Because the number of studies of tumor-infiltrating lymphocytes analyses was small, we didn’t test its publication bias and sensitivity analyses.

## Discussion

As an immune checkpoint, the PD-1/PD-L1 pathway plays an important role in regulating the expression of PD-L1 on immune cells, preventing T-cell mediated immune response, and regulating T-cell activation, proliferation, and cytokine release. Immune checkpoint inhibitors have been effective in the treatment of Hodgkin lymphoma and advanced melanoma [37]. PD-L1 is also an important target for immunotherapy. Nivolumab, a PD-1 blocking antibody, has been approved as a second-line treatment for HCC in patients who fail to respond to sorafenib treatment [38].

In the previous meta-analysis, Li et al. [39] pointed out the association between PD-L1 overexpression and worse OS and DFS. However, they failed to include sufficient studies. Additionally, it mistakenly regarded sPD-L1 and mPD-L1 as the same indicators. Two studies in this meta-analysis, Zeng et al. [32] and Finkelmeier et al. [33], adopted the ELISA method to test the level of sPD-L1, which were different from other studies, and those studies measured the expression of mPD-L1 in the tumor tissue, so the value of its pooled analysis was limited. In another meta-analysis by Liu et al. [40], the authors demonstrated that mPD-L1 expression had no correlation with OS and RFS, which may have resulted from the error in HR extraction from the Kaplan–Meier curves or the combining of RFS and DFS as the same outcome. Besides, it did not further explore the role of sPD-L1 in the prognosis of HCC patients.

In our meta-analysis, 23 articles were included, involving 3529 patients. We divided them into two subgroups. We grouped the studies where the mPD-L1 expression was determined in tumor cells using the IHC method together. In this group, we found significant correlation between high expression of mPD-L1 and worse OS. However, no significant correlation of mPD–L1 expression with DFS and RFS was found. High expression of mPD-L1 correlated with high AFP levels, history of hepatitis, and poor tumor differentiation. The high mPD-L1 expression positively correlated with CD8 + TILs, which confirmed the critical role that PD-L1 played in activating and proliferating T cells. In the other group, the soluble PD-L1 levels had significant correlation with poor OS. We used the HR reported in the article as much as possible for the pooled analysis. In studies were HR was not mentioned, it was extracted from the Kaplan–Meier curves by two investigators independently. Any disputes were further verified and discussed by the two investigators.

There was heterogeneity in our analysis due to several reasons. Taking the research by Gao et al. as an example [14], mouse anti-PD-L1 antibody was used for the IHC analysis; however, they did not distinguish the PD-L1 staining of HCC tumor cells in cell membrane and cytoplasm, and differentiated the ‘high’ and ‘low’ PD-L1 expression according to the density of staining. Other studies, such as that of Hu et al. [27] adopted a cutoff value of ≥1% tumor positive cells. The difference in the definition of mPD-L1 overexpression may be one of the sources of heterogeneity. At present, tumor positive cells ≥1% is the most widely accepted cutoff value for high expression [41], which was applied in the clinical trials of Nivolumab [38]. Most of the studies in the current meta-analysis used this cutoff value. Another reason for heterogeneity could be that the detection methods and antibodies were different in different studies. During the analysis between sPD-L1 and OS, we found that there were differences in the measurement methods of sPD-L1 in the included studies, which might have resulted in different cut-off values, leading to certain heterogeneity in our study.

In one of the studies by Chang et al. [25] with 1404 HCC patients involved, 1259 subjects underwent surgical resection alone while the remaining 145 patients were treated with surgery followed by post‐operative CIK cell immunotherapy (surgery‐CIK group). The results suggested that in surgery‐CIK group high expression of mPD-1 and tumor infiltrating lymphocytes (TILs) significantly correlated with OS and DFS, while in surgery‐only group, PD-L1 expression did not significantly correlate with OS. In another very similar study, a cohort study of 448 HCC patients (217 cases of hepatectomy alone and 231 cases of CIK cell transfusion after hepatectomy) revealed that the OS of PD-L1 positive patients in the combined treatment group was better, but no significant difference existed in the surgery-only group [19]. Based on the two studies, no association was observed between PD-L1 expression and OS in hepatectomy alone group. In tumor microenvironment, both tumor cells and immune cells could produce sPD-L1, so the sPD-L1 levels had only moderate correlation with the mPD-L1 expression in the tumor tissues [36]. Further studies are required to explore the role of sPD-L1 in the tumor immunity. We hypothesize that there may be an important role of PD-L1 expression in identifying patients likely to benefit from the immunotherapy for HCC.

In the present study, we found significant association between the soluble PD-L1 levels and OS. In clinical practice, only a small number of HCC patients undergo surgical resection, and liver biopsy is not routinely performed. Hence, obtaining tumor tissues for determining mPD-L1 expression by immunohistochemistry in clinical practice is difficult. On the contrary, determination of soluble PD-L1 is easier and showed a high prognostic value. Therefore, we recommend that future studies should focus on the prognostic role of soluble PD-L1 in HCC.

Although we systematically evaluated the relationship between PD-L1 expression and clinical features and prognosis, there still exist some deficiencies. First, studies from Asia accounted for majority of the included studies, thus probably limiting the results’ applicability to the rest of the world. Second, there was a lack of detailed analysis on the subsequent treatment after surgery. Most of the studies simply described the treatment after surgery and did not consider the differences between the treatments while making the final analysis. Finally, a few studies lacking original data made us unable to analyze the relationship between mPD-L1 expression and the clinical features in detail, such as the TNM stage, tumor size, cirrhosis, vascular invasion etc.

## Conclusions

The present meta-analysis supported that mPD-L1 overexpression in tumor tissues was highly associated with poor OS, although the short comings found in studies included were true. Moreover, high expression of mPD-L1 was associated with high AFP levels, hepatitis history, poor tumor differentiation, and TILs. The soluble PD-L1 levels were also significantly associated with poor OS. PD-L1 may be useful as a prognostic biomarker for HCC in future.

## Abbreviations

95% CI: 95% confidence interval
AFP: alpha-fetoprotein
DFS: disease-free survival
HCC: hepatocellular carcinoma
HR: hazard ratio
mPD-L1: membrane-bound PD-L1
OS: overall survival
PD-L1: programmed death ligand-1
RFS: relapse-free survival
sPD-L1: soluble PD-L1
